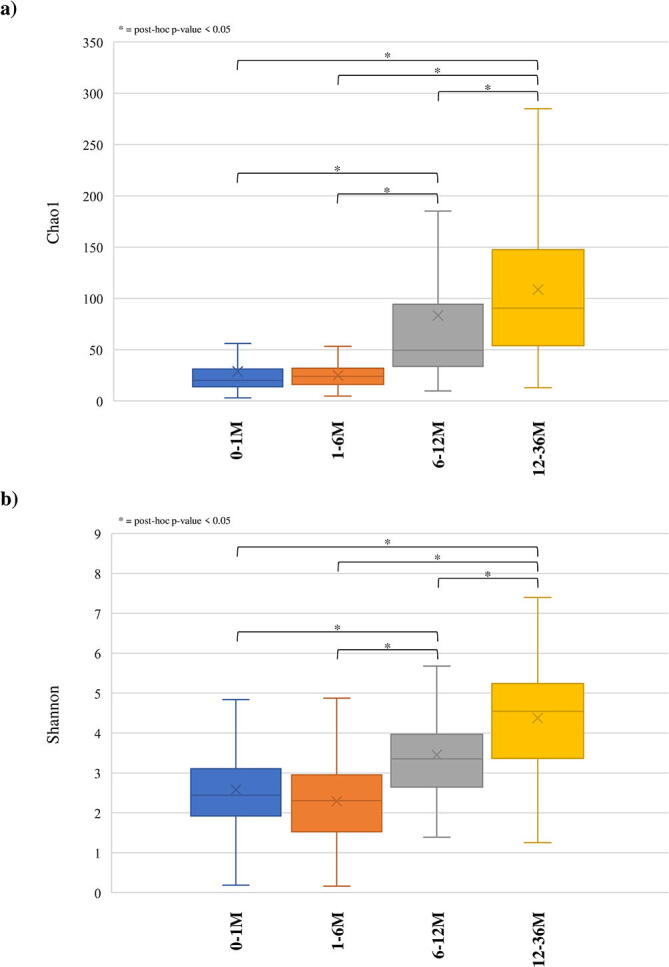# Multi-population cohort meta-analysis of human intestinal microbiota in early life reveals the existence of infant community state types (ICSTs)

**DOI:** 10.1016/j.csbj.2020.08.028

**Published:** 2020-09-15

**Authors:** Leonardo Mancabelli, Chiara Tarracchini, Christian Milani, Gabriele Andrea Lugli, Federico Fontana, Francesca Turroni, Douwe van Sinderen, Marco Ventura

**Affiliations:** a Laboratory of Probiogenomics, Department of Chemistry, Life Sciences and Environmental Sustainability, University of Parma, Parma, Italy; b APC Microbiome Institute and School of Microbiology, Bioscience Institute, National University of Ireland, Cork, Ireland

**Keywords:** Metagenomics, 16S rRNA profiling, Community state types

## Abstract

Appropriate development of the intestinal microbiota during infancy is known to be important for human health. In fact, aberrant alterations of the microbial composition during childhood may cause short- and/or long-term negative health effects. Many factors influence the initial assembly and subsequent progression of the gut microbiota of a neonate, such as feeding type, delivery mode, gestational age, maternal metabolic status and antibiotic exposure.

In the current study, the composition of the infant gut core-microbiota was explored, revealing particular variations of this core-microbiota during the first three years as influenced by delivery mode and feeding type. A multi-population cohort meta-analysis was performed by selecting 15 publicly available datasets pertaining to taxonomic profiles of 1035 fecal samples of healthy infants, as obtained by means of a 16S rRNA gene-based profiling approach. Interestingly, this multi-population cohort meta-analysis revealed great microbial complexity and specific taxonomic shifts in children older than six months, suggesting a major impact by the introduction of solid foods which prompts progression of infant gut microbiota towards that typical of adults. The taxonomic data sets employed in this multi-population cohort meta-analysis possess the statistical robustness to allow the identification of infant community state types (ICSTs). Our analysis therefore reveals the existence of specific taxonomic patterns that correspond to particular nutritional and developmental stages of early life, and that had previously been obscured by the high variability typical of such infant gut microbiota.

## Graphical abstract

**Figure:**
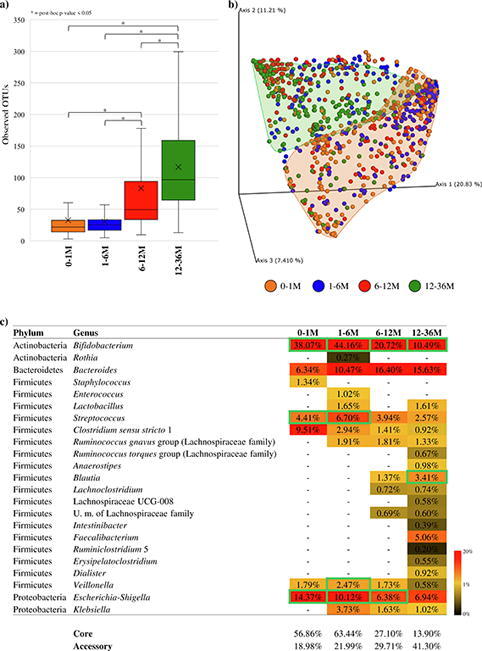

## 1 Introduction

The human gastrointestinal tract is colonized by billions of microorganisms, which are commonly referred to as the gut microbiota, and which plays a major role in maintaining host health by influencing and modulating nutritional, metabolic, immunological and physiological functionalities [1]. Consequently, initial gut colonization events and associated development of the gut microbiota in early (human) life is believed to elicit both temporary as well as long-lasting health effects, and to play a key role in defining host health status [2].

The early gut microbiota of healthy infants is characterized by low biodiversity and predominance of genera belonging to the Actinobacteria and Proteobacteria phyla, such as *Escherichia*, *Citrobacter* and *Bifidobacterium*, being influenced by several factors such as mode of delivery, diet and gestational age [1], [3]. With progressive age, the microbiota becomes more complex and bacteria belonging to Lachnospiraceae, Ruminococcaceae, Bacteroidaceae, Prevotellaceae, and Rikenellaceae families become the dominant taxa [4].

The delivery mode plays an important role in the establishment of the initial gut microbiota composition. In fact, comparison between natural and caesarean section (C-section) delivered infants has revealed several differences in their gut microbial population. In detail, the early gut microbiota of vaginally delivered infants has a composition enriched in vagina-associated microbes such as *Lactobacillus* and *Prevotella* [5], [6], while that of C-section delivered infants was shown to have reduced biodiversity, being characterized by the presence of environmental microorganisms that probably originate from maternal skin and hospital environment [7], [8].

The strong correlation between diet and development of the infant gut microbiota has been highlighted in several studies [9], [10]. Compared to formula-fed infants, breastfed infants have been shown to possess higher levels of bifidobacteria and lactobacilli [11], along with a lower abundance of the *Bacteroides* genus and potential pathogens belonging to clostridia, enterobacteria, enterococci and staphylococci [1].

The current study describes a multi-population cohort meta-analysis designed to evaluate the evolution of the infant microbiota composition during the early stages of life and its possible variations due to mode of delivery and diet. In particular, in order to define the composition of the infant core-microbiota from delivery up to the age of 3 years, we analyzed data based on a total of 1035 faecal samples of full-term, healthy infants. For this purpose, we selected 15 public datasets [12], [13], [14], [15], [16], [17], [18], [19], [20], [21], [22], [23], [24], [25], [26] obtained through 16S rRNA gene-based microbiota profiling, encompassing infants of an age that ranged between a few days to 3 years. Furthermore, we investigated variations of the infant gut microbiota based on the type of delivery and feeding of the newborn.

In contrast to the large part of the previously published meta-analyses regarding the human gut microbiota [27], [28], [29], [30], [31], [32], in our study we applied specific procedures aimed to minimize biases due to cross-study comparisons and maximize statistical accuracy. In detail, we selected metagenomic datasets that rely the same sequencing methodology and that are based on PCR primers with high taxonomic coverage. In this context, the taxonomic resolution of the different regions covered by the primer pairs included in this study was validated to ensure efficient comparison of the results. Furthermore, the raw data were re-analyzed using the same bioinformatics pipeline. Thus, our meta-analysis represents a relevant step-forward respect to currently available scientific literature, thereby allowing an in depth assessment of the microbial taxonomic patterns characterizing the gut microbiota development during infancy, and revealing the existence of distinct microbial assemblies named infant community state types (ICSTs).

## 2 Materials and methods

### 2.1 Database selection

In this meta-analysis, we obtained 1035 publicly available data sets from 15 studies involving the determination of the infant gut microbiota. In order to obtain high quality and coverage data, we selected 16S rRNA profiling datasets based on Illumina sequencing technology. The studies employed various primer pairs. In detail, we selected 16S rRNA microbial profiling datasets including only faecal samples from healthy infants with an age ranging between a few days to 3 years. No exclusions were made in the selected cohorts based on the mode of delivery and diet.

### 2.2 Evaluation of primer pair efficiency

The performance of the 11 primer pairs employed in the selected datasets was evaluated through the web-tool TestPrime 1.0 [33]. The latter executes an *in silico* PCR based on the SILVA database and provides the percentage of amplified sequences for each bacterial group at genus level [33]. The TestPrime was based on RefRN SILVA Database ssu-138 and a maximum of three mismatches was allowed [34].

### 2.3 16S rRNA profiling analysis

To avoid biases caused by different bioinformatic analysis pipelines, the sequence read pools of each dataset were filtered and analyzed through the same custom script based on the QIIME 2 software suite [35], [36]. Quality control maintained sequences with a length between 140 and 400 bp and average sequence quality score of >20, while sequences with homopolymers of >7 bp and mismatched primers were omitted. 16S rRNA Operational Taxonomic Units (OTUs) were defined at 100% sequence homology using DADA2 [37] and OTUs that were represented by just a single sequence were removed. All reads were classified to the lowest possible taxonomic rank using QIIME 2 [35], [36] and a reference dataset from the SILVA database v.132 [38]. In order to evaluate the bacterial biodiversity, alpha-diversity was assessed based on Good's coverage, Observed out, Chao1 and Shannon indexes and represented by box-and-whisker plots. Moreover, the Bray-Curtis dissimilarity index was used to estimate the beta-diversity between different age groups, delivery mode and diet. Dissimilarities were represented through a 3-Dimensional Principal Coordinate Analysis (PCoA).

### 2.4 Statistical analysis

QIIME 2 and SPSS software (www.ibm.com/software/it/analytics/spss/) were used to compute statistical analyses. PERMANOVA analyses were performed using 1000 permutations to estimate *p*-values for differences among populations in PCoA analyses. Furthermore, differential abundance of bacterial genera and alpha-diversity was tested by ANOVA analysis. Moreover, we also calculated the post hoc analysis LSD (least significant difference) for multiple comparison.

### 2.5 Infant community state type (ICST) prediction

The hierarchical clustering (HCL) of samples was obtained using bacterial composition at genus level and was calculated through TMeV 4.8.1 software using Pearson correlation as a distance metric based on information at genus level. The data obtained was represented by a cladogram.

## 3 Results and discussion

### 3.1 Selection of public datasets

The multi-population cohort meta-analysis performed in this study encompasses data sets corresponding to a total of 1035 faecal samples from healthy, full-term infants ranging from a few hours after birth to 3 years of age (Table 1 and Table S1). These 16S rRNA profiling datasets were selected so as to obtain a high sample number and reach corresponding robust statistical power, in accordance to previous studies [28], [29], [39]. In detail, we performed an in depth literature search for 16S rRNA profiling datasets based on Illumina sequencing technology, allowing us to retrieve microbiota data from 15 publicly available datasets covering 15 different countries (Table S1). Based on age at sampling, the datasets were subdivided into four macro-groups, i.e. 0–1 M group (<1 month of age), 1–6 M group (between 1 and 6 months of age), 6–12 M group (between 6 and 12 months of age) and 12–36 M group (between 1 and 3 years of age) (Table S1). This subdivision was made on the reported assumption that the infant gut microbiota is variable in composition and constantly evolving during the first three years after birth [40].

**Table 1:** Metadata of samples included in the meta-analysis.

Study (PMID)	Bioproject	Number of samples	Age (days) (mean ± st.dev)	Age (months) (mean ± st.dev)	Nation	Settlement	Socioeconomic status	Technology	Primer region
24896187	PRJEB5482	190	258 ± 233	8 ± 8	Bangladesh	Urban slum	low socioeconomic status	Illumina MiSeq	V1-V2
27160322	PRJNA318053	15	442 ± 262	15 ± 9	Ireland	Urban area	–	Illumina_MiSeq	V4–V5
27306664	PRJEB14529	225	246 ± 207	8 ± 7	USA	Urban area	–	Illumina MiSeq	V4
27717398	PRJNA331150	50	322 ± 104	11 ± 3	USA	Urban area	–	Illumina MiSeq	V4
28079170	PRJEB15633	18	115 ± 34	4 ± 1	Gambia	Urban area	–	Illumina_MiSeq	V4
28095889	PRJNA339264	167	8 ± 38	0 ± 0	Ireland	Urban area	–	Illumina MiSeq	V4–V5
28733284	PRJNA377056	9	383 ± 3	12 ± 0	Italy	Urban area	middle socioeconomic status	Illumina_MiSeq	V3-V4
28789705	PRJNA362530	10	28 ± 6	1 ± 0	Spain	Urban area	–	Illumina_MiSeq	V3
28877893	PRJEB21196	64	118 ± 51	4 ± 2	Germany	Urban area	–	Illumina_MiSeq	V3-V4
29184093	PRJNA403824	47	25 ± 31	1 ± 1	Canada	Urban area	–	Illumina MiSeq	V3
29217369	PRJEB21946	44	70 ± 0	2 ± 0	India	Densely populated Urban area	–	Illumina MiSeq	V4
29795809	PRJEB11419	127	646 ± 266	22 ± 9	USA Thailand United Kingdom Australia Ireland Norway Slovakia Canada	Urban area	–	Illumina MiSeq	V4
29884786	PRJNA450946 PRJNA450998 PRJNA451090 PRJNA451108 PRJNA451156 PRJNA451314 PRJNA451320 PRJNA451359 PRJNA451398 PRJNA451432 PRJNA453125 PRJNA451346	24	186 ± 182	6 ± 6	Norway	Urban area	–	Illumina HiSeq 2500	V4
30131575	PRJNA350676	20	387 ± 0	13 ± 0	Italy	Urban area	–	Illumina MiSeq	V3-V4
30579350	PRJNA491825	25	806 ± 215	27 ± 7	Denmark	Urban area	–	Illumina MiSeq	V3-V4

### 3.2 Homogeneity of the samples

16S rRNA gene-based microbiota profiling is the most commonly employed approach to perform gut microbiota profiling. This approach has many advantages, i.e. low cost, high sensitivity and specificity in the identification of bacterial taxonomy and availability of user-friendly bioinformatic pipelines, all of which have promoted its wide-spread application. Despite these advantages, there is extensive methodological variability, with subsequent output biases that may preclude consistent and meaningful comparisons between datasets obtained from different studies [41]. In particular, one of the main reasons for variable results is that different studies have employed different PCR primer pairs to amplify hypervariable region(s) of the 16S rRNA gene [41]. In order to evaluate the accuracy of the 11 primer pairs employed in the selected datasets (Table S2), we tested the primer pairs through the web-tool TestPrime 1.0 [33], [42]. TestPrime allows evaluation of the performance of a given primer pair by running an *in silico* PCR on the SILVA databases. Notably, this analysis showed rather similar amplification performances suggesting homogeneity of the selected datasets (Table S2). In particular, all tested primers showed an *in silico* efficiency of >95% in their ability to amplify the targeted 16S rRNA gene sequences (Table S2). While *in vitro* validation may provide additional evidences, these findings suggest the absence of any major bias in sequencing data generation. In order to confirm this result, we generated artificial communities constituted by genomes of species representative of the bacterial communities typically found in adult and infant feces (Table S3 and Table S4) [43]. These artificial communities were exploited to obtain PCR amplicons of the primer pairs included in this study and the data sets were processed using the bioinformatics pipeline based on the QIIME 2 software suite, as reported in material and methods section. Remarkably, the results obtained revealed that the profiles generated by the PCR primers pairs included in this study are comparable at the genus level, while, as expected, misclassifications may be observed at species level (Table S3 and Table S4). Thus, highlighting the homogeneity in microbial taxonomic efficiency/accuracy of the PCR primer pairs included in this genus-level meta-analysis. In addition, an identical bioinformatics pipeline based on the QIIME 2 software suite was employed for re-analysis of all the samples in order to allow a reliable and robust cross-meta-analysis of all datasets produced by these different research projects.

### 3.3 Meta-analysis of full-term infants’ microbiota over time

A total of 1035 publicly available datasets from 15 cohorts corresponding to faecal samples of full-term infants were retrieved. These combined cohorts were then divided in 285, 271, 215 and 264 samples belonging to the 0–1 M, 1–6 M, 6–12 M and 12–36 M groups, respectively (Table S1). Quality filtering resulted in a total of 40,014,085 reads with an average of 38,661 ± 48,855 per sample (Table S1). Evaluation of the Good's coverage index resulted in an average value of 0.99 ± 0.006, thereby indicating that sequencing depth of the assessed samples is adequate for a thorough analysis of the gut microbiota [44] (Table S5).

**Fig. 1:**
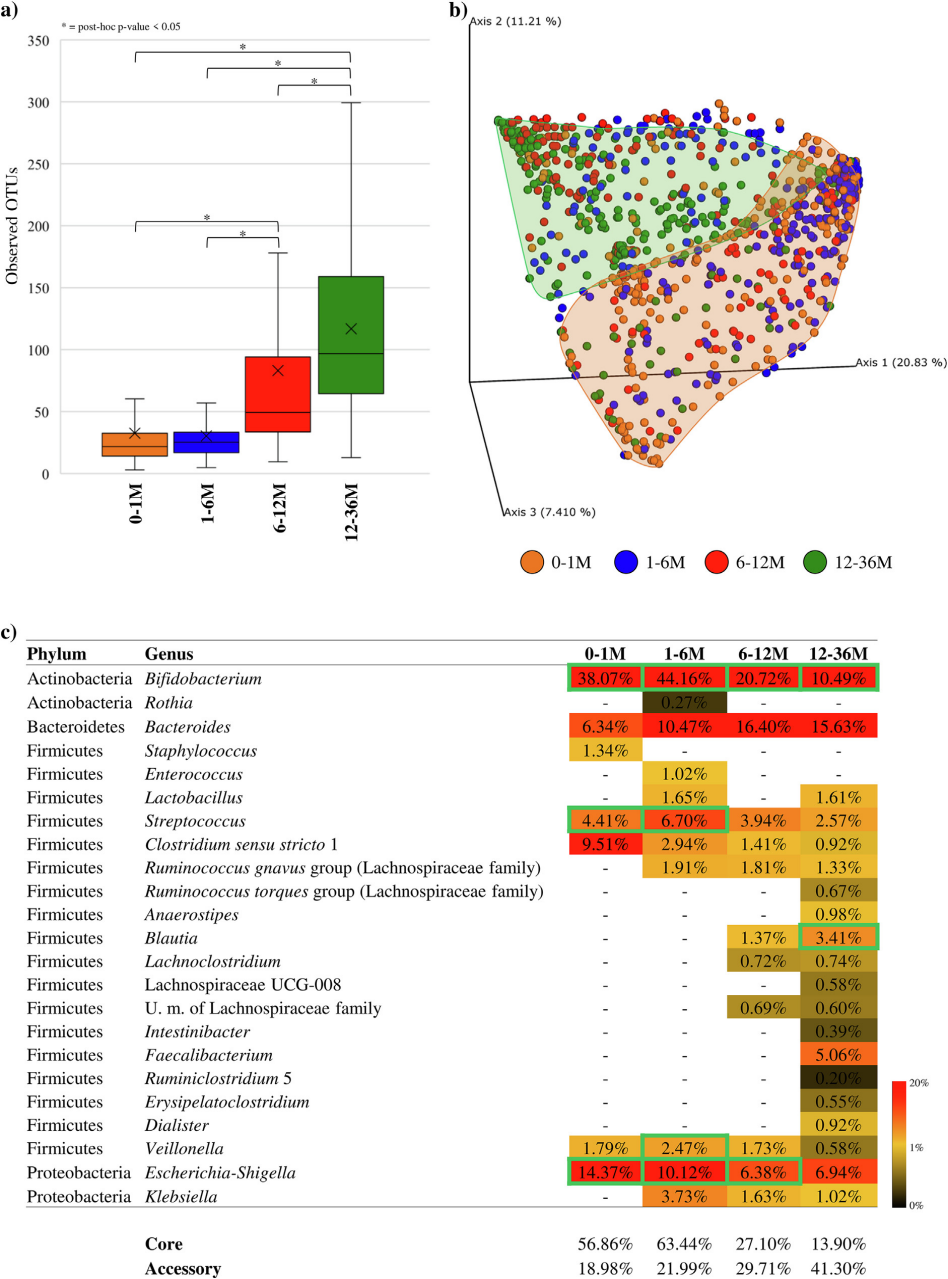
Exploration of the diversity and bacterial composition of healthy, full-term infant faecal samples over time. Panel a shows the box and whisker plot of the alpha-diversity calculated through Observed OTU index of the four age groups. The bottom and top of the box represent the first and third quartiles, and the band inside the box is the median. Moreover, the ends of the whiskers represent the minimum and maximum of all data of the sample. Panel b reports the principal coordinate analysis (PCoA) of the collected infant samples of the four age groups. Panel c displays the bacteria belonging to core- and accessory-microbiota of each age group. The genera belonging to the core are highlighted with a green outline. (For interpretation of the references to colour in this figure legend, the reader is referred to the web version of this article.)

Alpha-diversity analysis calculated through the Observed OTU, Chao1 and Shannon indexes showed statistically supported complexity differences between samples belonging to each of the four age groups (Fig. 1 and Figure S1). In fact, the 0–1 M and 1–6 M groups showed a statistically significantly lower biodiversity when compared to that of the 6–12 M and 12–36 M groups (ANOVA post-hoc *p*-value < 0.05 with Observed OTU, Chao1 and Shannon indexes) (Fig. 1a). These data support the notion that the infant gut microbiota gradually diversifies until weaning and subsequently develops towards a microbiota similar to that of adults [45], [46], [47], [48]. Remarkably, these results also underline the marked impact of weaning in shaping the evolution of the gut microbiota.

Analysis of the beta-diversity represented through Principal Coordinate Analysis (PCoA) based on Bray-Curtis dissimilarity revealed clear compositional differences between the four age-based groups (Fig. 1b). In particular, most of the samples belonging to 0–1 M and 12–36 M age sub-groups cluster separately (PERMANOVA *p*-value of < 0.001), while samples belonging to the 1–6 M and 6–12 M groups were shown to exhibit high inter-variability. Such findings indicate that the microbiota is constantly changing over time. These results were statistically validated by a pairwise PERMANOVA *p*-value of < 0.001.

In order to identify the main taxonomic differences between the gut microbiota of the 0–1 M, 1–6 M, 6–12 M and 12–36 M age-differentiated groups, taxonomic profiling at genus level was employed to reconstruct the core elements of the gut microbiota of each group [49]. This so-called core-microbiota was obtained by selecting bacterial genera that occur with a prevalence>70% among collected samples (of a particular age group) in at least 70% of the studies (Tables S6, S7, S8 and S7). Moreover, bacterial genera present in at least 70% of the studies and with a prevalence ranging from 30% to 70% were used to reconstruct the accessory-microbiota (Tables S6, S7, S8 and S9). The cut-off values were chosen based on previous publications [50], [51] and adapted to the high variability of the infant microbiota. Interestingly, the achieved results revealed that the core-microbiota (taxa with prevalence > 70%) of 0–1 M and 1–6 M groups was mostly represented by the *Bifidobacterium* genus (average abundance of 38.07% ± 34% and 44.16% ± 29.10%, respectively) (Fig. 1c, Tables S6 and S7). Conversely, the core-microbiota of 6–12 M and 12–36 M groups showed a relative decrease in this bacterial genus (average relative abundance of 20.72% ± 22.41% and 10.49% ± 15.81%, respectively) (*p*-value < 0.05) and a gradual increase in the core microbial complexity (Fig. 1c, Tables S8 and S9) characterized by the presence of *Bacteroides, Feacalibacterium, Blautia* and *Ruminococcus* genera (*p*-value < 0.05) (Fig. 1c). Furthermore, analysis of the data sets corresponding to the infant group younger than six months showed that the taxa with prevalence >70% in at least 70% of the studies, i.e. core-microbiota, represents on average of 60.07% ± 29.95% of the total identified bacterial community, while in infants aged between 12 and 36 months the core-microbiota comprises on average 27.19% ± 26.68% of the total bacterial community. Consequently, during the first 36 months following birth the gut microbiota of a newborn undergoes a progressive increase in accessory bacterial genera with prevalence ranging from 30% to 70%, corresponding to a reduction in the total relative abundance of the core microbiota.

### 3.4 Birth delivery mode

**Fig. 2:**
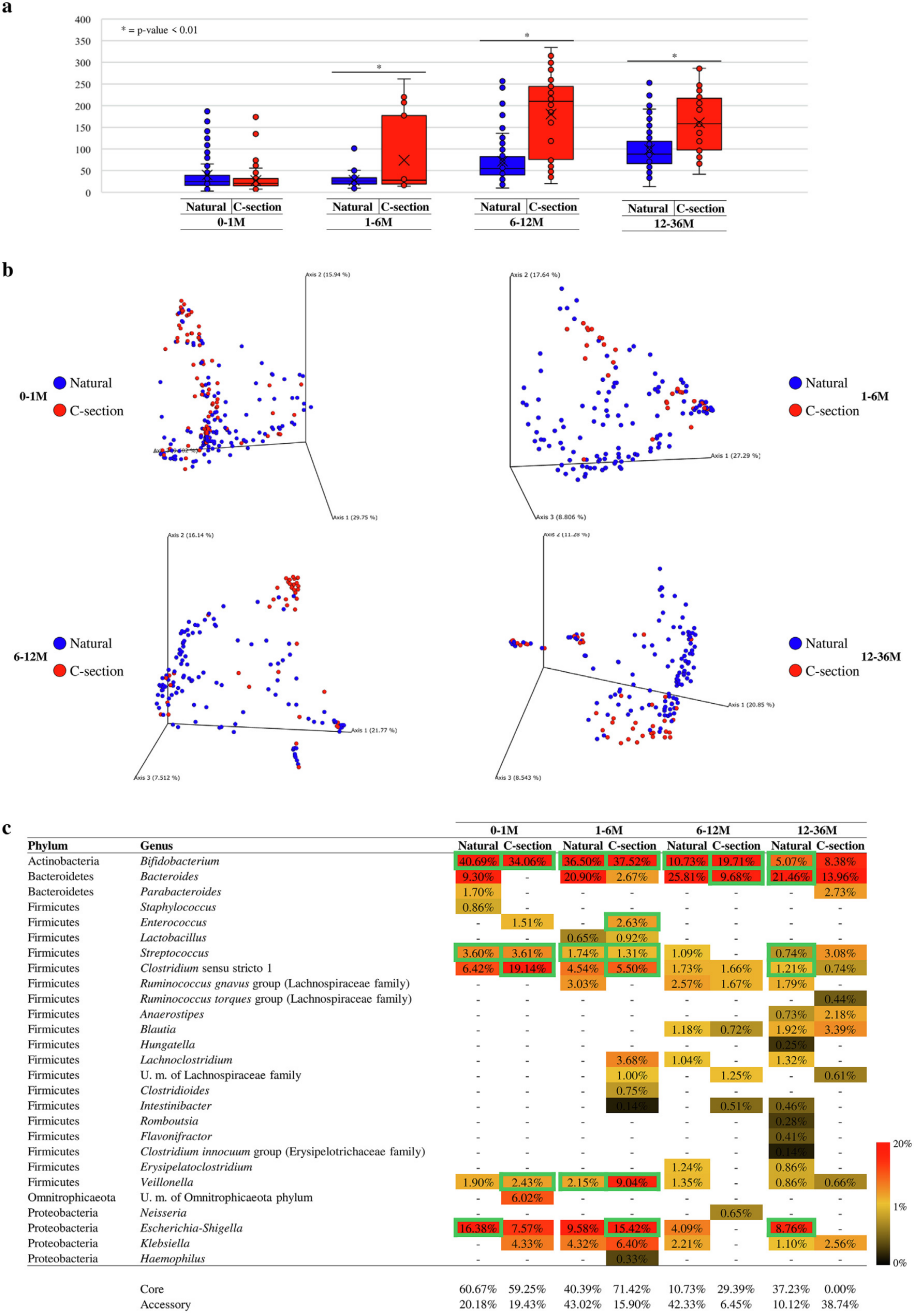
Evaluation of the diversity and bacterial composition of healthy, full-term infant faecal samples as based on birth delivery mode. Panel a indicates the box and whisker plot of the infant alpha-diversity calculated through Observed OTU index based on birth delivery methods. The bottom and top of the box represent the first and third quartiles, and the band inside the box is the median. Moreover, the ends of the whiskers represent the minimum and maximum of all data of the sample. Panel b reports the principal coordinate analysis (PCoA) of the collected infant samples, respectively, subdivided by age and birth delivery mode. Panel c displays the bacteria belonging to core- and accessory-microbiota of each delivery mode groups. The genera belonging to the core are highlighted with a green outline. (For interpretation of the references to colour in this figure legend, the reader is referred to the web version of this article.)

Several studies have highlighted the impact of delivery mode on the infant microbiota composition [1]. In order to identify possible differences between subjects born by natural or Caesarean delivery (C-section), samples within each age-differentiated group were further divided according to delivery method (Tables S10, S11, S12, S13, S14, S15, S16 and S17). Evaluation of microbial diversity through alpha-diversity (Fig. 2a) showed significant differences in 1–6 M, 6–12 M and 12–36 M age groups (*p*-value 0.01). In detail, the gut microbiota of infants born by C-section was shown to elicit a greater microbial complexity compared to vaginally born babies in these different age-based sub-groups (Fig. 2a). This variation can be explained by the contact of C-section newborns with environmental bacteria, resulting from contact of the baby with the mother's skin and hospital environment, which will colonize and affect the composition of the infant gut microbiota [52]. Furthermore, lack of exposure of the newborn to the mother's vaginal/rectal microbiota may cause reduced mother-to-baby bacterial transfer, and allow skin/hospital-associated microbes to colonize in the absence of competition [1].

Analysis of beta-diversity, based on Bray-Curtis dissimilarity and represented through PCoA plots, revealed a spatial division based on delivery mode for all four age sub-groups. Furthermore, these results were confirmed by PERMANOVA analysis (*p*-value < 0.01) (Fig. 2b).

Scrutiny of the bacterial composition of babies born by C-section indicated a microbiota characterized by the presence of microorganisms that typically colonize hospital environments and the human skin. In detail, C-section delivered infants belonging to the 0–1 M group revealed a 197.98% higher abundance of *Clostridium* sensu stricto 1 genus compared to those born vaginally (*p*-value < 0.05) (Table S18). Similarly, a 265.78% (*p*-value < 0.01) higher abundance of the *Escherichia-Shigella* genus was observed in subjects born by C-section within the 6–12 M group (Table S18). In contrast, infants born by natural delivery showed in all age groups a higher abundance of the *Bacteroides* genus compared to C-section delivered infants, being 149.91%, 682.14%, 166.64% and 53.74% more abundant in 0–1 M, 1–6 M, 6–12 M and 12–36 M groups, respectively (*p*-value 0.01) (Table S18). As highlighted in previous studies, the lower abundance of *Bacteroides* in infants born by C-section (compared to naturally delivered infants), with a corresponding increase in other taxa, may contribute to an increased risk in the development of inflammation and obesity [14], [53], [54], [55]. Focusing on the *Bifidobacterium* genus, one of the most representative bacteria in all four age groups, significant differences are observed only in the 6–12 M group (Table S18) suggesting rather minor, if any impact of the type of delivery on this bacterial genus.

### 3.5 Influence of feeding on the infant microbiota

**Fig. 3:**
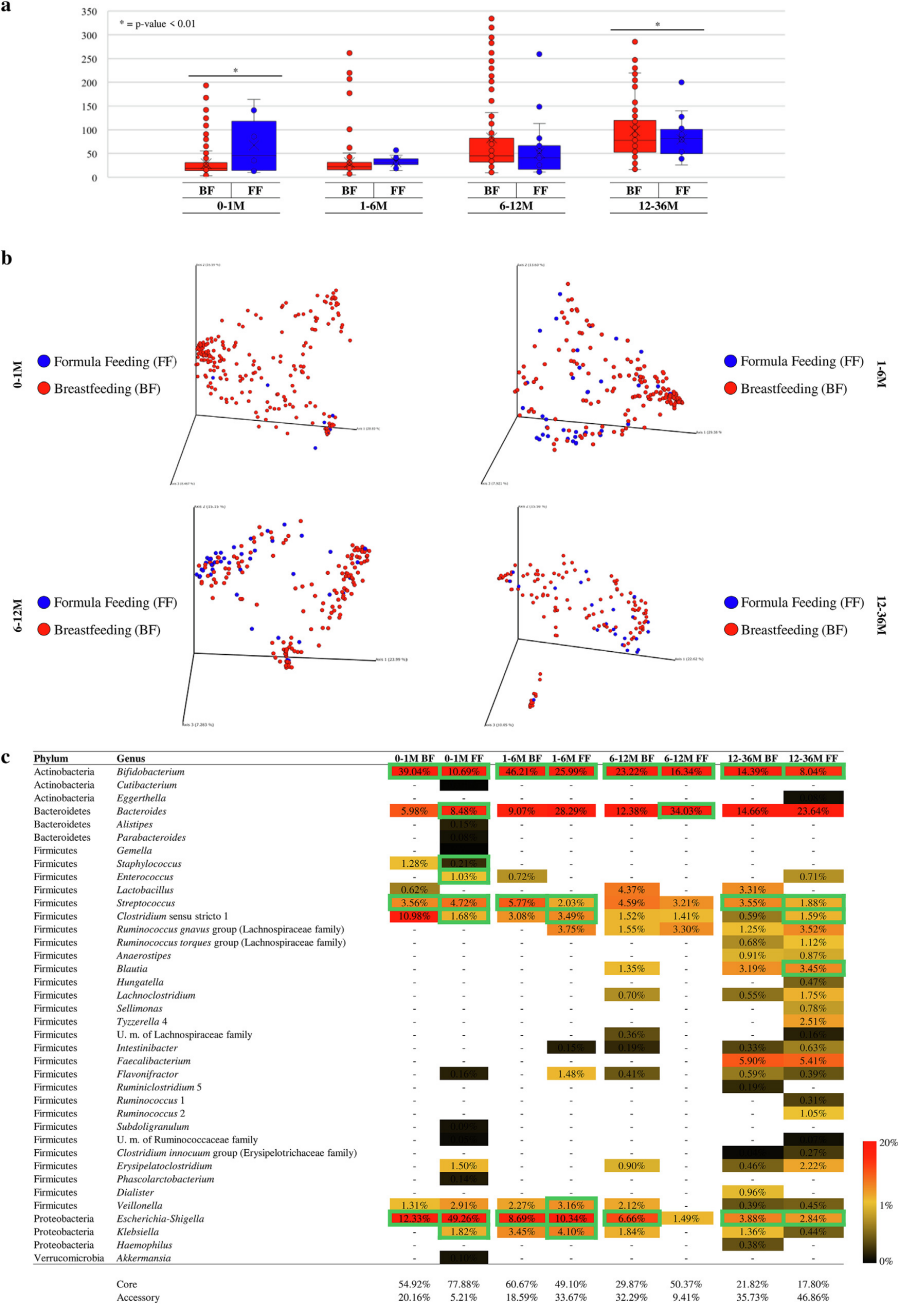
Examination of the diversity and bacterial composition of healthy, full-term infant faecal samples based on type of feeding. Panel a shows the box and whisker plot of the infant alpha-diversity calculated through Observed OTU index based on type of feeding. The bottom and top of the box represent the first and third quartiles, and the band inside the box is the median. Moreover, the ends of the whiskers represent the minimum and maximum of all the data of the sample. Panel b reports the principal coordinate analysis (PCoA) of the collected infant samples, respectively subdivided by age and type of feeding. Panel c displays the bacteria belonging to core- and accessory-microbiota of each age group. The genera belonging to the core are highlighted with a green outline. (For interpretation of the references to colour in this figure legend, the reader is referred to the web version of this article.)

Breastfeeding, as opposed to the use of formula milk, is known to modulate the gut microbial population. In fact, the oligosaccharides present in human milk (i.e. human milk oligosaccharides or HMOs) cannot be digested by the infant itself and are metabolized by certain bacteria present in the infant gut, as these bacteria possess and express the necessary genetic repertoire to allow HMO metabolism, thus facilitating the selection of specific intestinal bacterial species [56]. In order to highlight possible differences between breastfeeding and formula feeding, samples within each age-differentiated group were divided by type of feeding (Tables S19, S20, S21, S22, S23, S24, S25 and S26). Evaluation of alpha-diversity based on the Observed OTU index did indeed reveal significant microbiota compositional differences between breastfed babies compared to infants fed with formula milk, but such differences reached statistical significance only in the 0–1 M and 6–12 age groups (*p*-value < 0.01) (Fig. 3a). These results indicates that the complexity of the microbiota, at least at genus level, is not strictly related to the feeding type, but is more likely to be subject to major changes by the transition to a semi-solid/solid diet that typically occurs at an average age of 6 months [57], [58]. Biodiversity analysis revealed significant differences between the breastfed babies compared to infants fed with formula milk in the four distinct age groups (PERMANOVA < 0.05) (Fig. 3b).

In order to identify possible differences in gut microbiota composition between breastfed and formula-fed infants for the four defined age-based macro-groups, the taxonomic changes at genus level were analyzed. This analysis showed that breastfed children possess higher levels of the *Bifidobacterium* genus in their gut microbiota when compared to age-matched children fed with formulated milk, with significant differences in all age groups (*p*-value < 0.01) (Table S27). Furthermore, the *Escherichia-Shigella* genus showed an average relative abundance of 13.38% ± 22.85% in breastfed infants of 0–1 M group, while subjects fed with formula milk revealed an average relative abundance of 35.25% ± 37.32% (*p*-value < 0.01). Additionally, significant differences have been highlighted for the genus *Bacteroides*, which showed the highest levels of relative abundance in the babies fed with formula milk. In detail, comparisons between breastfed and formula-fed infants showed a significant absolute difference (*p*-value < 0.01) of 19.22%, 21.26% and 16.83% in 1–6 M, 6–12 M and 12–36 M groups, respectively. These results show that the gut microbiota of breastfed infants when compared to that of infants nourished with formula milk possess a higher relative abundance of *Bifidobacterium* [59], confirming the importance of breast milk intake in modulating the gut microbiota during the early stages of life toward presumed health-promoting microbial taxa [60].

### 3.6 Identification of ICSTs

In 2011 Arumugam et al. [48] proposed that the gut microbiota of adults can be stratified into three distinct clusters driven by discriminative genera. These three clusters, also called enterotypes, include *Bacteroides* (enterotype 1), *Prevotella* (enterotype 2), and *Ruminococcus* (enterotype 3) [48]. Furthermore, a recent study performed an analysis of the enterotypes of children at school-going age by identifying three distinct clusters, dominated by *Bacteroides*, *Prevotella*, and *Bifidobacterium*, respectively [61]. Moreover, a longitudinal study on neonatal gut suggested the possible existence of community state types (CSTs) characterized by the presence of Enterobacteriaceae, *Veillonella*, *Ruminococcus*, *Streptococcus*, *Prevotella*, *Bacteroides*, and *Bifidobacterium* [62]. Consequently, despite the extreme dynamism of the infant gut microbiota that had previously prevented identification of taxonomic patterns and cluster-discriminative genera, we exploited the statistical robustness offered by our extensive meta-analysis to investigate the possible existence of infant community state types (ICSTs) that correspond to the early stages of life. In this context, the distinctly higher microbial gut community complexity of infants aged between 12 and 36 months when compared to that of children less than six months old (see meta-analysis discussed above), prompted us to divide the samples into two macro groups, i.e. a 0–6 M group (<6 months) and a 6–36 M group (>6 months). Interestingly, these two macro groups seem to correspond to the division between pre- and post-weaning infants indicated by the guidelines of the World Health Organization (WHO) [58] and reported in previous studies [57].

**Fig. 4:**
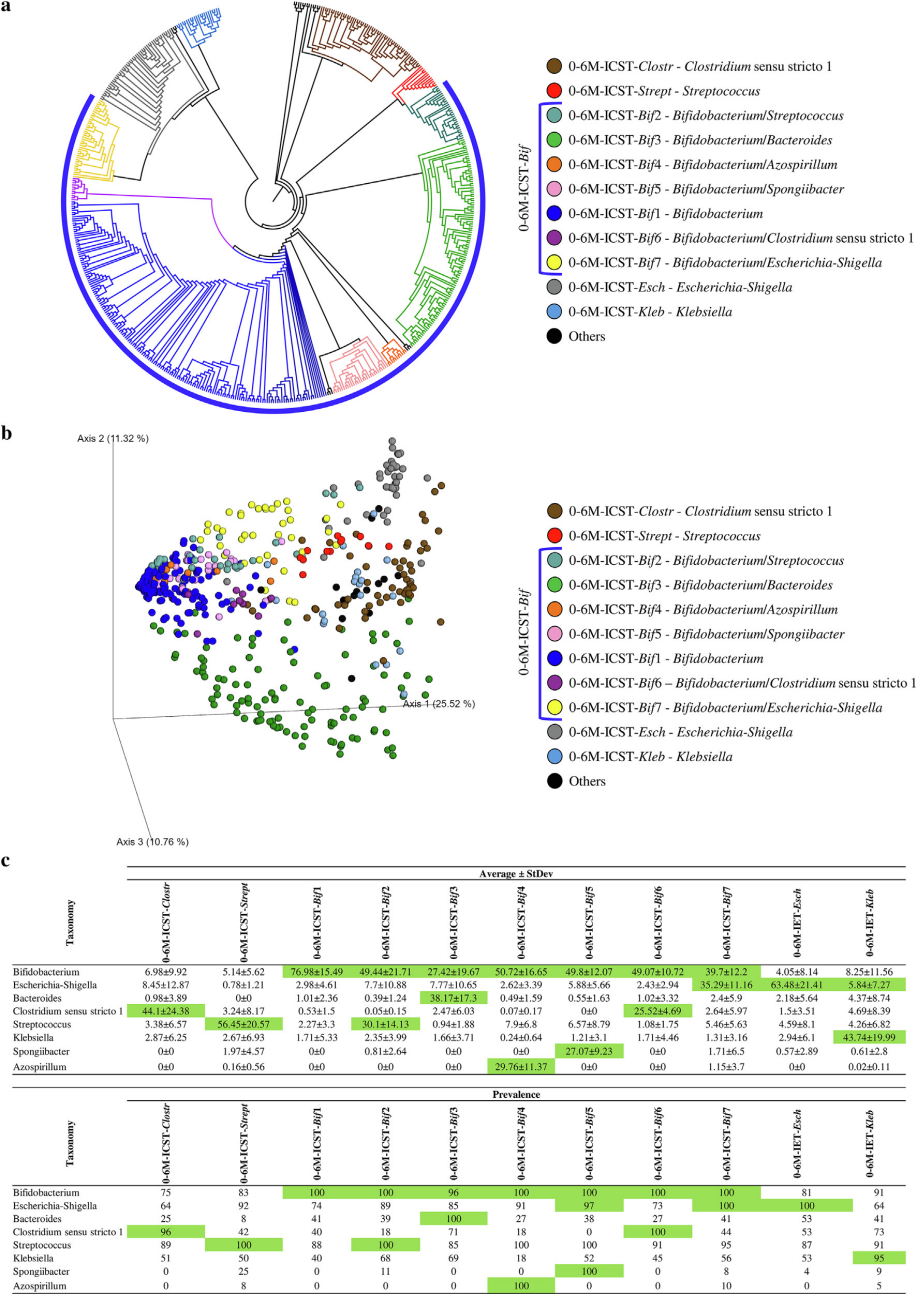
Identification of 0–6 M group infant ICSTs. Panel a shows a circular cladogram of the samples belonging to the 0–6 M group, obtained by means of hierarchical clustering (HCL) analysis. Panel b reports the principal coordinate analysis (PCoA) of the 0–6 M infant samples, subdivided by ICST. Panel c displays the average abundance and prevalence of bacteria that correspond to an identified ICST. Values that represent the ICSTs cut-off are highlighted in green. (For interpretation of the references to colour in this figure legend, the reader is referred to the web version of this article.)

**Fig. 5:**
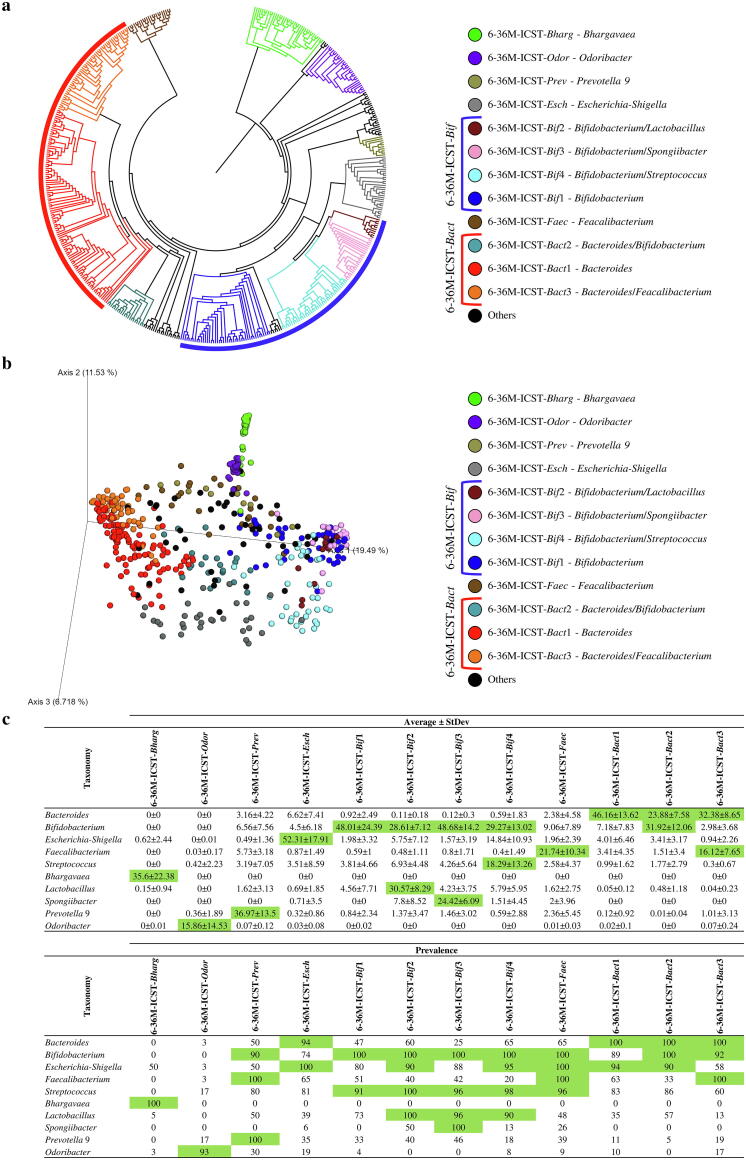
Identification of infant ICSTs based on the microbiota of faecal samples associated with the 6–36 M group. Panel a illustrates a circular cladogram of the samples belonging to the 6–36 M group, based on hierarchical clustering (HCL) analysis. Panel b shows the principal coordinate analysis (PCoA) of the 6–36 M samples, subdivided by ICSTs. Panel c reports the average abundance and prevalence of the bacteria that correspond to an identified ICST. The values that represent the ICST cut-off are highlighted in green. (For interpretation of the references to colour in this figure legend, the reader is referred to the web version of this article.)

Screening for ICSTs present in these two groups was performed by cluster analysis through hierarchical clustering (HCL) (Fig. 4a and 5a) and 3D Bray Curtis PCoA (Fig. 4b and 5b) of the collected samples as based on their 16S rRNA gene-derived microbial profiles (Tables S28 and S29). Each statistically supported cluster had to be represented by at least 10 samples to be considered as a putative ICST. Moreover, the representative taxa of each proposed ICST had to be present at an average abundance and prevalence higher than 15% and 90%, respectively (Fig. 4c). Results were verified by PCoA analyses followed by statistical validation through PERMANOVA (*p*-value < 0.05).

Analysis of the 0–6 M samples allowed us to identify five main infant community state types (0–6 M-ICST-*Clostr*, -*Strept*, -*Bif*, -*Esch* and -*Kleb*) (Fig. 4), while samples from the 6–36 M macro-group revealed seven main ICSTs (6–36 M-ICST-*Bharg*, -*Odor*, -*Prev*, -*Esch*, -*Bif*, -*Faec* and -*Bact*) (Fig. 4). Interestingly, the high number of detected ICSTs is presumed to reflect the high variability and dynamism of the microbiota at the early stages of life [63]. Particularly, the 0–6 M-ICSTs were shown to be dominated by the *Bifidobacterium* genus (0–6 M-ICST-*Bif)*, which is present with an abundance average higher than 15% in 70% of the samples (Fig. 4a). In detail, 174 samples of this bifidobacteria-dominated ICST (0–6 M-ICST-*Bif*) contained high levels of members of the *Bifidobacterium* genus (0–6 M-ICST-*Bif*1, bifidobacterial average abundance of 76.98%), while the 224 remaining samples were characterized by the combination of *Bifidobacterium* genus with other bacterial taxa (0–6 M-ICST-*Bif*2, -*Bif*3, -*Bif*4, -*Bif*5, -*Bif*6 and -*Bif*7) (Fig. 4a). Conversely, the 6–36 M groups showed a decrease in community state types characterized by the presence of the genus *Bifidobacterium* (6–36 M-ICST-*Bif* main community state types encompassing 24.84% of the samples) and an increase of ICSTs characterized by the genus *Bacteroides* (6–36 M-ICST-*Bact* main community state types encompassing 37.16% of the samples) (Fig. 5a). The community state type characterized by the dominance of the *Bacteroides* genus (6–36 M-ICST-*Bact*1) and ICSTs dominated by typical adult bacterial genera, such as *Feacalibacterium* (6–36 M-ICST-*Faec*), highlight the shift of the infant’s microbiota towards a bacterial community similar to that of adults.

Previous studies have reported that ethnicity in the early stages of life plays a major role in the evolution of the gut microbiota [64], [65], [66]. Since the metadata accompanying the 16S rRNA microbial profiling data used in this study do not provide any detail about ethnicities, we decided to investigate possible correlations between the metagenomic data and the geographical origins of the samples. Analysis of the 0–6 M samples revealed that most nations are characterized by high prevalence of 0–6 M-ICST-*Bif* (prevalence in each nation >30%) except for the Spanish samples that seem most represented by 0–6 M-ICST-*Esch* (Table 2). Conversely, samples from the 6–36 M macro-group showed heterogeneity in the distribution of ICSTs. In detail, Danish, German, Norwegian and US samples seem to be characterized by high prevalence of 6–36 M-ICST-*Bact* (>30%), while Italian and English samples showed prevalence >30% of 6–36 M-ICST-*Bif*1 and 6–36 M-ICST-*Odor*, respectively (Table 3). These results seem to underline a possible correlation between geographical origin and infant gut microbiota composition, especially in samples older than 6 months probably due to the transition to a solid diet. A specific study regarding geographical origin, race and socioeconomic status categories of infants could provide a much sharper view of their role in the maturation of the gut microbiome.

**Table 2:** Evaluation of distribution of 0–6 M-ICSTs in nations included in the study. 0–6 M-ICSTs with a presence greater than 30% are highlighted in green.

**Table 3:** Assessment of distribution of 6–36 M-ICSTs in nations included in the study. 6–36 M-ICSTs with a presence greater than 30% are highlighted in green.

In addition, we evaluated co-variance between the bacterial genera with a total average abundance of > 0.5%. In detail, co-variance analysis based on 0–6 M group samples (Table S30) revealed that the *Bifidobacterium* genus negatively correlates with most other taxa that dominate in non-bifidobacterial ICSTs, such as *Clostridium* sensu stricto 1 (0–6 M-ICST-*Clostr*), *Streptococcus* (0–6 M-ICST-*Strept*), *Escherichia*-*Shigella* (0–6 M-ICST-*Esch*) and *Klebsiella* (0–6 M-ICST-*Kleb*). Similarly, analysis of samples corresponding to 6–36 M infants showed a negative correlation between the *Bifidobacterium* and *Bacteroides* genera, which constitute the most representative community state types, yet revealed a positively correlation between typical adult taxa, such as *Bacteroides*, *Faecalibacterium* and *Alistipes* (Table S31). These results support the notion that during infancy *Bifidobacterium* represents the most dominant genus and is replaced during weaning by bacteria typical of an adult microbiota.

### 3.7 Evaluation of ICSTs corresponding to delivery mode and feeding type

The infant microbiota composition appears to be predominantly influenced by the delivery mode and feeding type (see above). We therefore decided to evaluate if and how ICSTs may be modulated by these two factors. This analysis as based on 0–6 M group samples showed that infants born by vaginal delivery were mainly represented by the 0–6 M-ICST-*Bif*1 (31.16% of the total samples) and 0–6 M-ICST-*Bif*3 (33.70% of the total samples) community state types , which are dominated by the *Bifidobacterium* genus (Table 4). Similarly, samples from babies born by C-section stratified within the bifidobacterial community state type 0–6 M-CST-*Bif*1 (33.63% of the total samples), yet were also assigned to the community state type 0–6 M-ICST-*Clostr* (29.20% of the total samples) were dominated by the environmental *Clostridium* sensu stricto 1 (Table 4). Interestingly, samples from infants born by natural delivery and belonging to the 6–36 M group were shown to be frequently assigned to the *Bacteroides* community state types 6–36 M-ICST-*Bact*1 (37.07% of the total samples) and 6–36 M-ICST-*Bact*3 (12.07% of the total samples), while C-section infants were not associated with a dominant ICST except for 6–36 M-ICST-*Bif*4 (23.33% of the total samples), which is characterized by *Bifidobacterium* and *Streptococcus* genera (Table 5).

**Table 4:** Evaluation of the ICSTs based on delivery.

	n° samples	0–6 M-ICST-*Clostr*	0–6 M-ICST-*Strept*	0–6 M-ICST-*Bif*1	0–6 M-ICST-*Bif*2	0–6 M-ICST-*Bif*3	0–6 M-ICST-*Bif*4	0–6 M-ICST-*Bif*5	0–6 M-ICST-*Bif*6	0–6 M-ICST-*Bif*7	0–6 M-ICST-*Esch*	0–6 M-ICST-*Kleb*
Natural	276	6.88%	1.09%	31.16%	1.81%	33.70%	0.00%	0.00%	2.54%	5.07%	9.06%	3.26%
C-section	113	29.20%	1.77%	33.63%	0.00%	8.85%	0.00%	0.00%	3.54%	3.54%	8.85%	7.08%
Breastfeeding (BF)	393	11.70%	1.78%	32.32%	3.56%	17.30%	2.80%	7.38%	2.54%	4.58%	7.89%	4.58%
Formula Feeding (FF)	45	2.22%	2.22%	11.11%	0.00%	53.33%	0.00%	0.00%	0.00%	8.89%	15.56%	4.44%

**Table 5:** Valuation of the ICSTs based on feeding.

	n° samples	6–36 M-ICST-*Bharg*	6–36 M-ICST-*Odor*	6–36 M-ICST-*Prev*	6–36 M-ICST-*Esch*	6–36 M-ICST-*Bif*1	6–36 M-ICST-*Bif*2	6–36 M-ICST-*Bif*3	6–36 M-ICST-*Bif*4	6–36 M-ICST-*Faec*	6–36 M-ICST-*Bact*1	6–36 M-ICST-*Bact*2	6–36 M-ICST-*Bact*3
Natural	229	9.91%	7.76%	1.29%	6.03%	1.72%	0.00%	0.00%	2.16%	5.17%	37.07%	6.90%	12.07%
C-section	89	15.56%	11.11%	1.11%	11.11%	3.33%	3.33%	0.00%	23.33%	0.00%	15.56%	5.56%	4.44%
Breastfeeding (BF)	264	10.07%	5.97%	1.49%	2.99%	8.96%	3.36%	7.84%	11.57%	6.72%	19.03%	5.97%	7.09%
Formula Feeding (FF)	65	7.58%	1.52%	1.52%	3.03%	4.55%	1.52%	4.55%	3.03%	3.03%	48.48%	6.06%	12.12%

Focusing on the correlation between infant community state types and the mode of feeding, the 0–6 M breast-fed samples were shown to be frequently assigned to the community state types 0–6 M-ICST-*Bif*1 (32.32% of the total samples) or 0–6 M-ICST-*Bif*3 (17.30% of the total samples) being characterized by dominance of the *Bifidobacterium* and *Bacteroides* genera, respectively, while samples from formula-fed infants were most commonly assigned to the community state type 0–6 M-ICST-*Bif*3 (53.33% of the total samples) (Table 4). Instead, the 6–36 M breast-fed samples revealed a high heterogeneity in the distribution of ICSTs, while samples from formula-fed babies showed a prevalence of the *Bacteroides* community state type 6–36 M-ICST-*Bact*1 (48.48% of the total samples) (Table 5). These results confirm the impact of the feeding type, i.e. breast- and formula-feeding, on the infant microbiota, suggesting a close correlation between formula-fed babies and the *Bacteroides* ICST.

## 4 Conclusions

The human gastrointestinal tract is colonized by millions of microorganisms collectively defined as microbiota. In this context, several studies have shown that appropriate development of the intestinal microbiota during the early stages of life may positively influence human health.

In order to investigate changes in the intestinal microbiota during the early stages of life in human, we performed a multi-population cohort meta-analysis of a total of 1035 faecal samples of healthy, full-term infants ranging from a few hours following birth to 3 years of age. The multi-population cohort meta-analysis revealed a higher microbial complexity in samples from infants aged between 12 and 36 months compared to those from children less than one month old. This increase in biodiversity appears to have caused a reduction in the abundance of the *Bifidobacterium* genus in favor of *Bacteroides*, *Feacalibacterium*, *Blautia* and *Ruminococcus* genera. The multi-population cohort meta-analysis allowed us to observe higher complexity in subjects born by C-section compared to those born by natural delivery. In addition, babies born by C-section were associated with a bacterial colonization characterized by genera commonly present in the environment and/or bacterial genera that may be pathogenic. The multi-population cohort meta-analysis also revealed a positive correlation between breastfeeding and dominance of bifidobacteria in the corresponding gut microbiota.

The statistical robustness offered by our extensive meta-analysis allowed us to identify infant community state types (ICSTs) highlighting that healthy children less than six months old are characterized by the predominance of the ICST with high relative abundance of the *Bifidobacterium* genus in their gut microbiota, while infants aged between 12 and 36 months are characterized by typically adult bacterial genera, such as *Bacteroides* and *Feacalibacterium*. Moreover, analysis of ICSTs highlighted a strong correlation between formula-fed infants and the development and maintenance of a microbiota with a predominance of the *Bacteroides* genus. Certainly, a deep meta-analysis of datasets based on whole-metagenome shotgun (WMS) or built on internal transcribed spacer (ITS)-based profiling, will offer a more detailed species level investigation and will no doubt allow the confirmation of these infant community state types described here and the discovery of further ICSTs or sub-ICSTs. Furthermore, our results highlight the need for additional studies accompanied by accurate metadata that will be crucial in order to precisely determine the factors that influence the evolution of the infant gut microbiota. Moreover, due to the absence of longitudinal studies involving metagenomic data across the 3 years, it was not possible to evaluate if these identified ICSTs remain stable over time. In this context, a specific longitudinal study spanning a wider age range may be important to clarify the evolution and stability of the microbiota in relation to the identified community state types and highlight possible correlations between the ICSTs of infants and adults.

## CRediT authorship contribution statement

**Leonardo Mancabelli:** Conceptualization, Methodology, Formal analysis, Investigation, Data curation, Visualization, Writing - original draft. **Chiara Tarracchini:** Methodology, Investigation, Data curation, Visualization. **Christian Milani:** Methodology, Supervision, Writing - review & editing. **Gabriele Andrea Lugli:** Conceptualization, Software. **Federico Fontana:** Data curation. **Francesca Turroni:** Conceptualization, Supervision. **Douwe Sinderen:** Conceptualization, Supervision, Writing - review & editing. **Marco Ventura:** Conceptualization, Supervision, Project administration, Writing - review & editing.

## Declaration of Competing Interest

The authors declare that they have no known competing financial interests or personal relationships that could have appeared to influence the work reported in this paper.

## Figures and Tables

**Supplementary figure 1:**